# Supplementary material for: Impact of Dietary Modifications on Plasma Sirtuins 1, 3 and 5 in Older Overweight Individuals Undergoing 12-Weeks of Circuit Training
Source: Nutrients. 2021 Oct 27;13(11):3824. doi: 10.3390/nu13113824 (PMC8624957; doi:10.3390/nu13113824)
Supplement: Supplementary file 1 [file nutrients-13-03824-s001.zip › nutrients-1399043-SI.pdf]

nutrients

Supplementary

Impact of Dietary Modifications on Plasma Sirtuins 1, 3 and 5 in Older Overweight Individuals Undergoing 12-Weeks of Circuit Training

Paulina Wasserfurth[1 2], Josefine Nebl[1], Miriam Rebekka Rühling[3], Hadeel Shammas[3], Jolanthe Bednarczyk[3], Karsten Koehler[2] , Tim Konstantin Boßlau[3], Karsten Krüger[3], Andreas Hahn[1] and Anibh Das[3*]

Table S1: Name and sequence of qRT-PCR-Primers.

Primer	Sequence
human SUPT20H (forward)	CAC CAA CCC CTT CAT CGA CC
human SUPT20H (reverse)	CCT GGC TCA GAG ACC TTT GC
human SIRT1 (forward)	CAA CTT GTA CGA CGA AGA C
human SIRT1 (reverse)	TCA TCA CCG AAC AGA AGG
human SIRT3 (forward)	CAG TCT GCC AAA GAC CCT
human SIRT3 (reverse)	AAA TCA ACC ACA TGC AGC AA
human SIRT5 (forward)	AGT GGT GTT CCG ACC TTC AG
human SIRT5 (reverse)	CAT CGA TGT TCT GGG TGA TG

Table S2. Dietary intakes from 3-day food logs.

		CON	EX	EXDC	EXCO	*p*
Energy intake (kcal/day)	pre	1792 ± 322	2028 ± 483	1955 ± 527	1917 ± 541	0.501
	post	1906 ± 293	1814 ± 378	1634 ± 528*	1858 ± 417	0.548
	Δ	114 ± 435	-214 ± 436	-322 ± 422	-59 ± 674	0.215
Protein (%E/day)	pre	18 ± 2	16 ± 4	17 ± 4	16 ± 4	0.629
	post	18 ± 2	17 ± 4	18 ± 4	16 ± 3	0.387
	Δ	-0.11 ± 2	0.66 ± 4	2 ± 4	-0.11 ± 5	0.722
Fat (%E/day)	pre	36 ± 6	37 ± 9	41 ± 7	39 ± 7	0.340
	post	40 ± 7	38 ± 7	39 ± 9	36 ± 8	0.722
	Δ	4 ± 8	1 ± 8	-2 ± 7	-3 ± 8	0.283
CHO (%E/day)	pre	41 ± 6	42 ± 11	37 ± 5	40 ± 7	0.125
	post	37 ± 7	40 ± 5	37 ± 7	41 ± 7	0.357
	Δ	-4 ± 7	-2 ± 9	-0 ± 7	1 ± 4	0.191
Fiber (g/day)	pre	19 ± 8	24 ± 8	20 ± 9	23 ± 7	0.384
	post	19 ± 11	19 ± 7*	22 ± 5	26 ± 13	0.227
	Δ	0 ± 7	-5 ± 7	2 ± 9	3 ± 10	0.083
EPA (g/day)	pre	0.24 ± 0.65	0.11 ± 0.17	0.03 ± 0.02	0.28 ± 0.64	0.841
	post	0.04 ± 0.03	0.16 ± 0.30	0.09 ± 0.07	0.07 ± 0.07	0.767
	Δ	-0.20 ± 0.64	0.05 ± 0.36	0.06 ± 0.08	-0.21 ± 0.66	0.241
DHA (g/day)	pre	0.25 ± 0.41	0.21 ± 0.21	0.18 ± 0.11	0.28 ± 0.31	0.829
	post	0.12 ± 0.06	0.28 ± 0.46	0.21 ± 0.12	0.16 ± 0.17	0.538
	Δ	-0.13 ± 0.38	0.07 ± 0.51	0.03 ± 0.23	-0.12 ± 0.31	0.903
Retinol equivalent (µg/day)	pre	1252 ± 846	1478 ± 731	1486 ± 736	1846 ± 2217	0.627
	post	1315 ± 861	1299 ± 815	1617 ± 1064	1466 ± 566	0.672
	Δ	63 ± 968	-179 ± 1059	132 ± 1495	-380 ± 2227	0.892
Vitamin C (mg/day)	pre	93 ± 60	165 ± 102	116 ± 59	109 ± 44	0.138
	post	129 ± 115	106 ± 38*	153 ± 66	117± 77	0.277
	Δ	36 ± 81	-59 ± 108	37 ± 75	8 ± 61	0.058
α-Tocopherol (mg/day)	pre	7 ± 2	10 ± 4	11 ± 7	11 ± 5	0.105
	post	9 ± 2**	10 ± 4	36 ± 74	11 ± 5	0.899
	Δ	2 ± 2	-0 ± 6	25 ± 76	0. ± 6	0.618

Data are shown as mean ± SD. Differences among groups were assessed with Kruskal-Wallis-Test, differences within groups were-detected with Wilcoxon test (* p < 0.05; ** p < 0.01).

Table S3: Dietary intakes from food frequency questionnaires.

		CON	EX	EXDC	EXCO	*p*
Fruit intake (portion/ day)	pre	1.4 ± 1.1	1.7 ± 1.2	1.5 ± 1.2	1.8 ± 1.7	0.783
	post	1.4 ± 0.7	1.9 ± 2.3	2.5 ± 1.3*	1.4 ± 0.8	0.151
	Δ	0.0 ± 1.2	0.2 ± 1.3	1.0 ± 1.1	-0.4 ± 1.5	0.076
Vegetable in-take (portion/ day)	pre	1.8 ± 3.2	1.2 ± 0.8	0.9 ± 0.3	0.9 ± 0.8	0.719
	post	0.7 ±0.6*	1.0 ± 0.7	1.4 ± 0.8	1.0 ± 0.8	0.284
	Δ	-1.1 ± 2.7 **#**	-0.1 ± 0.5	0.6 ± 0.7 **#**	0.1 ± 1.0	**0.018**
Cereal intake (portion/ day)	pre	3.8 ± 2.4	3.0 ± 2.5	3.8 ± 2.9	3.4 ± 1.7	0.581
	post	2.4 ±0.8*	2.6 ± 1.4	2.9 ± 1.6	2.7 ± 1.4	0.983
	Δ	-1.4 ± 1.8	-0.5 ± 1.2	-0.9 ± 3.1	-0.6 ± 1.8	0.643
Green & black tea (cups/ day)	pre	0.3 ± 0.5	1.5 ± 2.2	0.5 ± 0.8	2.2 ± 3.1	0.582
	post	1.1 ± 1.5	0.9 ± 1.7	2.2 ± 3.0	0.6 ± 1.0	0.932
	Δ	0.7 ± 1.3	-0.6 ± 2.5	1.6 ± 3.4	-1.6 ± 3.3	0.232
Coffee (cups/ day)	pre	4.7 ± 7.9	2.6 ± 4.6	3.7 ± 2.1	2.8 ± 2.1	0.152
	post	1.4 ± 1.3	2.5 ± 1.7	2.8 ± 3.3	2.5 ± 1.3	0.490
	Δ	-3.2 ± 8.3	-0.1 ± 4.5	-0.9 ± 4.2	-0.4 ± 2.4	0.561
Wine & spar-kling wine (glases/ day)	pre	0.3 ± 0.2	0.4 ± 0.6	0.2 ± 0.2	0.5 ± 0.4	0.406
	post	0.2 ± 0.2	0.7 ± 1.1	0.2 ± 0.4	0.2 ± 0.2	0.192
	Δ	-0.0 ± 0.2	0.3 ± 0.7	0.1 ± 0.4	-0.2 ± 0.4	0.097

Data are shown as mean ± SD. Differences among groups were assessed with Kruskal-Wallis-Test, differences within groups were detected with Wilcoxon test (*p < 0.05; ** p < 0.01; # p < 0.001)

.
